# A Rare Case of Systemic Sarcoidosis in a Middle-Aged Female with a Solitary Thyroid Nodule

**DOI:** 10.1155/2021/5231703

**Published:** 2021-12-21

**Authors:** Ayrton Bangolo, John Bukasa Kakamba, Ali Atoot, Mohammad Jurri, Ashraf Mahmoud, Arthur Oliver Lo, Kunchang Song, Syed Sirajuddin, Adam Atoot

**Affiliations:** ^1^Department of Internal Medicine, Hackensack Meridian Health Palisades Medical Center, North Bergen, NJ, USA; ^2^Endocrine and Metabolic Disorders Department, University Clinics of Kinshasa, Kinshasa, Democratic Republic of the Congo; ^3^Department of Endocrinology, University of Liege, Liege, Belgium; ^4^Department of Anesthesia, Hackensack University Medical Center, Hackensack, NJ, USA; ^5^Department of Family Medicine, Hackensack Meridian Health Palisades Medical Center, North Bergen, NJ, USA; ^6^University of the East Ramon Magsaysay, Quezon City, Philippines; ^7^Department of Pathology, Hackensack Meridian Health Palisades Medical Center, North Bergen, NJ, USA

## Abstract

Sarcoidosis is a multisystem inflammatory disease characterized by the presence of noncaseating granulomas. Sarcoidosis can affect any organ of the body, the lung being the most affected. Sarcoidosis rarely affects the thyroid gland, and only a few cases of thyroid-related sarcoidosis have been reported in the literature. Herein, we report a case of systemic sarcoidosis with multiple organ involvement in a patient with a solitary left thyroid nodule and benign Fine Needle Aspiration (FNA) findings. The patient was eventually diagnosed with thyroid sarcoidosis using a core needle biopsy of the thyroid nodule. With this case report, we highlight the limitations of the FNA. This case report has the objective to encourage clinicians to include thyroid sarcoidosis in the differential diagnosis of thyroid nodules in patients with systemic sarcoidosis even with unremarkable FNA findings.

## 1. Introduction

Sarcoidosis is a multisystem granulomatous disorder of unknown etiology that affects individuals worldwide and is characterized pathologically by the presence of noncaseating granulomas in involved organs [1]. Up to 30 percent of patients present with extrapulmonary sarcoidosis [2]. Approximately 8 percent of patients with sarcoidosis present with disease at extrapulmonary sites without lung involvement. In these patients, the skin is the most common site and accounted for nearly half of the patients [3]. Thyroid involvement results in diffuse goiter or solitary thyroid nodule [4]. Most patients with thyroid involvement remain euthyroid and only a few cases have been associated with hypothyroidism [5]. We report the case of a middle-aged female who presented for evaluation of hypercalcemia and was found to have systemic sarcoidosis and a solitary left thyroid nodule. The thyroid nodule was found to be unremarkable on Fine Needle Aspiration (FNA), but sarcoid infiltration of the gland was revealed on Core Needle Biopsy. With this case report, we aim to encourage physicians to include sarcoidosis in the differential diagnosis of thyroid nodules even with normal FNA and recognize the limitations of FNA.

## 2. Case Presentation

**Figure 1:**
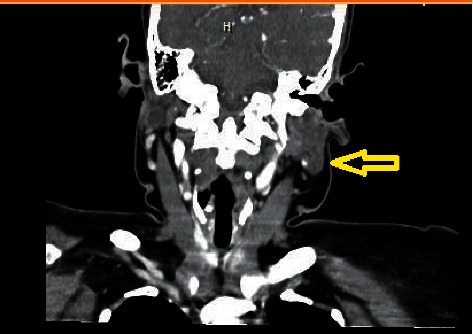
Neck soft tissue computed tomography revealing the left thyroid nodule.

This is a 58-year-old female with a past medical history of Diabetes Mellitus and asthma who presented for evaluation of hypercalcemia. The patient was referred from the Primary Care Physician's office after routine blood work revealed elevated calcium levels. Of note, the patient visited her primary care physician for evaluation of cough and dyspnea one month prior; encounter during which a chest roentgenogram revealed left lobe enlargement with right tracheal deviation. She underwent a subsequent computed tomography (CT) of neck soft tissue which revealed 3.5 centimeters nodule of the left thyroid gland (Figure 1). A fine needle aspiration of the nodule revealed a Bethesda category II, benign. However, the specimen was marginally adequate. She was also diagnosed with hypothyroidism and levothyroxine was initiated. She reported a globus sensation for the past 2 months prior to our encounter. She also reported abdominal pain associated with constipation for the past week prior to our encounter. She denied any dysphagia or change in voice.

**Figure 2:**
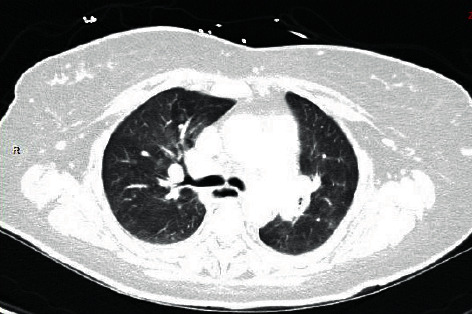
Chest computed tomography revealing mediastinal and hilar lymphadenopathy.

**Figure 3:**
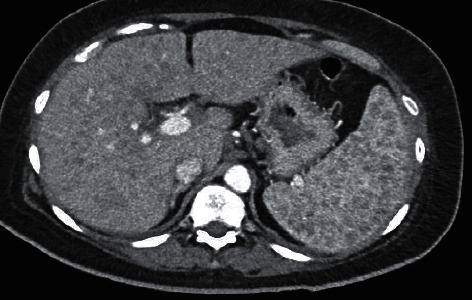
Computed tomography of the abdomen revealing multiple nodules of the liver and spleen.

Upon arrival in the Emergency Department, she was hemodynamically stable. A left thyroid nodule with cervical lymphadenopathy could be felt on physical examination. Her laboratory results revealed an elevated serum calcium, higher normal 1,25(OH)_2_ vitamin D and lower normal 25(OH) vitamin D, and an elevated Angiotensin Converting Enzyme level, as seen in Table 1. A CT of the chest and abdomen revealed mediastinal and hilar lymphadenopathy (Figure 2) and multiple nodules of the liver and spleen (Figure 3). A subsequent biopsy of the liver revealed granulomatous hepatitis consistent with sarcoidosis.

**Figure 4:**
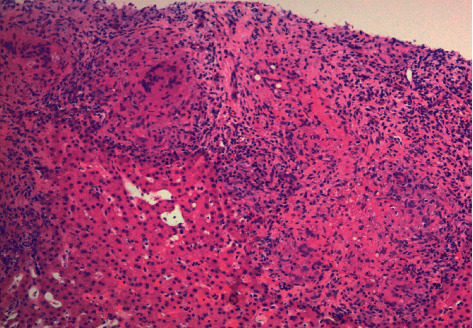
Thyroid nodule core biopsy pathology report revealing non-necrotizing granulomatous infiltration consistent with sarcoidosis. Trichrome stain showed no significant fibrosis, and iron stain was negative for iron deposition.

The patient was started on normal saline continuous infusion. Nephrology and oncology were consulted. The patient's calcium level had normalized on day 2 of hospitalization. She eventually underwent a core needle biopsy of the left thyroid nodule which revealed granulomatous infiltration of the gland consistent with sarcoidosis (Figure 4).

She was referred to Ear, Nose, and Throat for a thyroidectomy given the invasive nature of the nodule.

## 3. Discussion

Sarcoidosis is an inflammatory disease characterized by the presence of noncaseating granulomas. Although sarcoidosis can affect virtually every organ of the body, the lung is most affected. Other organs commonly affected are the liver, skin, and eye [6]. Sarcoidosis rarely affects the thyroid gland. Sarcoidosis can cause diffuse goiter or rarely solitary or multiple thyroid nodules [4] that resemble a malignancy, especially when associated with cervical adenopathy [7]. Although most patients are euthyroid, both hypothyroidism and hyperthyroidism have been reported [8]. Our patient's sarcoidosis has multiple organs involved including the thyroid gland, lymph nodes, liver, spleen, lungs, and mediastinum. She has a solitary thyroid nodule with lymphadenopathy; those findings can mimic a thyroid cancer with metastasis.

Respiratory complaints including cough and dyspnea are the most common presenting symptoms. Fatigue is the most common constitutional symptom [6]. The most common abnormality of liver function is an elevation of the alkaline phosphatase level, consistent with an obstructive pattern. In addition, elevated transaminase levels can occur [6]. Our patient was initially complaining of cough and dyspnea, but a chest roentgenogram failed to show characteristic features of sarcoidosis which delayed the diagnosis. She did not have any constitutional symptoms. The liver profile revealed a cholestatic pattern consistent with sarcoidosis. Hypercalcemia occurs in about 10% of sarcoidosis patients. The mechanism of abnormal calcium metabolism is increased production of 1, 25-dihydroxyvitamin D by the granuloma itself [6]. As seen in our patient, hypercalcemia was associated with higher normal 1, 25(OH)_2_ vitamin D and a lower normal 25(OH) vitamin D. Several conditions can cause mild elevation of Angiotensin Converting Enzyme (ACE) but only a few conditions, including sarcoidosis, will cause an ACE elevation more than 50% of the upper limit of normal [6]. Elevated levels of ACE are reported in 60% of patients with acute disease and only 20% of patients with chronic disease [6]. Our patient had an ACE level elevation of more than 50% of the upper limit of normal, consistent with sarcoidosis.

Thyroid sarcoidosis is diagnosed with fine needle aspiration (FNA) biopsy or thyroidectomy for treatment of goiter demonstrating noncaseating epithelioid granulomas [9]. The false-negative rate of a benign interpretation is 0 to 3 percent [10]. In a retrospective study of nodules with two or more prior nondiagnostic FNAs, core needle biopsy was diagnostic in 86 compared with 29 percent for repeat FNA [11]. Core needle biopsy has low nondiagnostic result rates and high specificity for the diagnosis of malignancy. It is a safe diagnostic technique with a higher diagnostic yield, especially when molecular testing is not available or fine needle aspiration does not yield enough cells for molecular testing [12]. Our patient underwent an initial FNA with a marginally adequate cellularity; thus, a definitive diagnosis could not be obtained. A repeat FNA was warranted but given the higher specificity and sensitivity of the core needle biopsy compared to a repeat FNA; our patient underwent a core needle biopsy. The procedure revealed the final diagnosis.

Patients with sarcoidosis of the thyroid do not usually need treatment unless they have a goiter of sufficient size to cause obstructive symptoms or thyroid dysfunction. Thyroidectomy is generally performed in patients with obstructive symptoms or other absolute indications for surgery, such as thyrotoxicosis or thyroid cancer. Hypothyroidism, if present, should be treated with thyroid hormone therapy [9]. Our patient has dyspnea and tracheal deviation on imaging caused by a large thyroid nodule; thus, she was referred for surgery. The large nodule also caused hypothyroidism for which she is on levothyroxine.

## 4. Conclusion

Sarcoidosis is a multisystem granulomatous disease that can affect any organ. Some organs are more affected than others. The thyroid gland is one of the least affected organs, and only a few cases of thyroid-related sarcoidosis have been reported in the literature. Fine needle aspiration (FNA) is an important diagnostic tool in the evaluation of thyroid nodules. However, FNA can have limitations in the diagnosis of certain conditions requiring more cells for molecular testing such as sarcoidosis. Core needle biopsy has low nondiagnostic results and should be used in case the FNA sample is marginally adequate. With this case report, we hope to contribute to the growing literature of thyroid-related sarcoidosis and encourage physicians to include such a condition in the differential of thyroid nodules even with unremarkable FNA.

## Figures and Tables

**Table 1:** Laboratory values on admission.

	Laboratory values
Calcium	**14** milligram/deciliter (mg/dl) (8.6–10.3)
Aspartate aminotransferase	**44** units/liter (U/L) (13–39)
Alanine aminotransferase	47 U/L (7–52)
Parathyroid hormone	**3** picogram/milliliter (pg/ml) (14–64)
Parathyroid hormone-related peptide	20 pg/ml (14–27)
25(OH) vitamin D	22.3 nanogram/milliliter (20–80)
1,25(OH)_2_ vitamin D	66 pg/ml (18–72)
Phosphorus	3.6 mg/dl (3.0–4.3)
Thyroid-stimulating hormone	2.87 milli-international units per milliliter (0.450–4.50)
Anti-thyroid peroxidase antibody	9 international units per milliliter (IU/mL) (0–34)
Anti-thyroglobulin antibody	<1.00 IU/mL (0.0–0.9)
Alkaline phosphatase (ALP)	**323** U/L (34–104)
Bone specific ALP	**29.6** microgram/milliliter (5.6–29)
Gamma-glutamyl transferase	**282** U/L (9–64)
Angiotensin-1-converting enzyme	**115** U/L (9–67)

## Data Availability

All data generated or analyzed during this study are available from the corresponding author upon request.
